# Diagnostic Challenges in Choroid Plexus Tumours

**DOI:** 10.1111/nan.70095

**Published:** 2026-07-20

**Authors:** Christian Thomas, Martin Hasselblatt

**Affiliations:** ^1^ Institute of Neuropathology University Hospital Münster Münster Germany

**Keywords:** choroid plexus carcinoma, choroid plexus papilloma, diagnostics, molecular classification, *TERT* sequencing, *TP53* sequencing

## Abstract

Choroid plexus tumours are rare epithelial neoplasms arising from the choroid plexus, accounting for approximately 0.2% of all central nervous system tumours but up to 20% of brain tumours diagnosed during the first year of life. CPTs exhibit marked clinical and biological heterogeneity and are classified into three entities according to the World Health Organization (WHO): choroid plexus papilloma (CPP, WHO grade 1), atypical choroid plexus papilloma (aCPP, WHO grade 2) and choroid plexus carcinoma (CPC, WHO grade 3). Although CPP is a benign tumour with excellent prognosis following complete surgical resection, aCPP is defined by increased mitotic activity and carries a higher risk of recurrence in children older than 3 years and in adults. In addition, a subset of adult (a)CPPs harbours *TERT* promoter mutations, which have been associated with an increased risk of tumour recurrence. CPC is a highly malignant tumour characterised by frank signs of malignancy and aggressive clinical behaviour, with particularly poor outcomes in *TP53*‐mutant cases, frequently associated with *Li–Fraumeni* syndrome. Accurate grading and distinction from histological mimics may be difficult, especially in small biopsy specimens or tumours with atypical features. In addition, the integration of molecular findings, including DNA methylation analysis and copy‐number assessment, has become an important adjunct in the diagnostic evaluation of CPTs by improving diagnostic accuracy and providing additional prognostic information. In this review, we outline the histological and molecular characteristics of CPTs and highlight common diagnostic pitfalls and relevant differential diagnoses across different age groups.

## Summary

- Choroid plexus tumours comprise choroid plexus papilloma, atypical choroid plexus papilloma and choroid plexus carcinoma, with mitotic activity serving as the most reproducible histological criterion for grading and recurrence risk.
- Accurate diagnosis requires awareness of age‐dependent differential diagnoses, including AT/RT, CRINET and medulloepithelioma/ETMR in infants; papillary ependymoma, teratoma and PTPR in children and adolescents; and ELST, PTPR and metastases in adults.
- Targeted immunohistochemistry supports the distinction of choroid plexus tumours from histological mimics, including SMARCB1/INI1, LIN28A, EMA, Kir7.1, stanniocalcin‐1, SPDEF and organ‐specific metastatic markers.
- DNA methylation profiling and copy‐number assessment can improve diagnostic confidence in challenging cases and provide additional prognostic information, particularly for atypical choroid plexus papilloma.
- *TERT* promoter mutations in adult CPP/aCPP and *TP53* alterations in CPC provide clinically relevant risk stratification; genetic counselling and germline *TP53* testing are recommended for all patients with CPC to identify Li–Fraumeni syndrome.

## Graphical abstract

In this review, we summarise the diagnostic spectrum of choroid plexus tumours, highlight key age‐dependent differential diagnoses across infants, children/adolescents and adults, and outline how histopathology, immunohistochemistry and molecular profiling support accurate tumour classification.

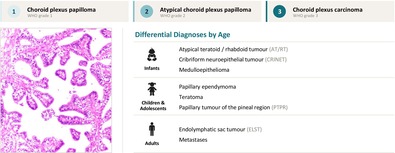

## 1 Introduction

Choroid plexus tumours (CPTs) are rare epithelial neoplasms derived from the choroid plexus, accounting for approximately 0.2% of all central nervous system (CNS) tumours but up to 20% of brain tumours diagnosed during the first year of life [1, 2]. The annual incidence is 0.4 per 1,000,000 population [3]. CPTs typically arise within the ventricular system, reflecting the normal distribution of choroid plexus tissue: the lateral ventricles (55%), third ventricle (11%) and fourth ventricle or cerebellopontine angle (34%) [4]. Rare ectopic locations such as the sellar and pineal regions or intraparenchymal sites have been reported [5, 6, 7]. The median age at diagnosis across all CPTs is 3.5 years, with significant variation by location: tumours of the lateral and third ventricles present at a median age of 1.5 years, whereas fourth ventricular tumours occur predominantly in adults (median age: 22.5 years) [8]. According to the current 2021 World Health Organization (WHO) classification, CPTs are stratified into three tumour types: choroid plexus papilloma (CPP, WHO grade 1), atypical choroid plexus papilloma (aCPP, WHO grade 2) and choroid plexus carcinoma (CPC, WHO grade 3). Accurate grading, integration of molecular findings and distinction from histological mimics may be difficult, particularly in small biopsy samples or tumours with atypical features. In this review, we describe the histological and molecular characteristics of CPTs and highlight common diagnostic pitfalls and relevant differential diagnoses across different age groups.

## 2 Histopathology and Diagnostic Criteria of CPTs

### 2.1 CPP (WHO Grade 1)

CPP is a benign papillary neoplasm closely resembling the normal choroid plexus architecture. It is composed of uniform cuboidal to columnar epithelial cells, often with basally located nuclei, lining delicate fibrovascular cores in a well‐formed papillary pattern. Compared with non‐neoplastic choroid plexus, tumour cells appear more crowded and lack the characteristic cobblestone appearance created by intercellular spaces. Mitotic activity is absent or minimal (≤ 1 mitosis per 10 high‐power fields; 0.23 mm^2^). Necrosis, increased cellularity, nuclear pleomorphism or loss of papillary architecture may occur in CPP, but the isolated presence of any of these features is not associated with higher recurrence risk [9]. Rare histological variants include oncocytic change, mucinous degeneration, melanisation, neuropil‐like islands, stromal deposition of elastic fibres and even mesenchymal differentiation such as formation of bone, cartilage or adipose tissue [10, 11]. Clinical outcome after gross total resection is excellent, with a 10‐year overall survival rate of 97% [12]. Recurrence is uncommon (approximately 6%), and malignant transformation to CPC is exceedingly rare [13]. Although dissemination can occur, metastatic disease is uncommon in CPP [14].

### 2.2 aCPP (WHO Grade 2)

aCPP was introduced in the 2007 WHO classification to address the limitations of the former binary distinction between CPP and CPC. This intermediate category was established after clinicopathological analyses demonstrated that increased mitotic activity was the only independent histological predictor of recurrence in CPP [9]. Accordingly, aCPP is defined as a CPP with increased mitotic activity (≥ 2 mitoses per 10 high‐power fields; 0.23 mm^2^). Additional atypical features such as increased cellularity, nuclear pleomorphism, focal blurring of the papillary pattern with solid growth or necrosis may be present but are not required for diagnosis. The prognostic significance of aCPP is age‐related because children aged > 3 years and adults are more likely to recur, whereas young children below the age of 3 years at diagnosis show a good prognosis that is comparable to CPP [15, 16]. The biological basis for this age‐related effect remains unclear but most likely reflects developmental differences in the proliferative milieu of the choroid plexus during early childhood.

### 2.3 CPC (WHO Grade 3)

In contrast to CPP and aCPP, CPC is a highly malignant tumour with frank signs of malignancy, often characterised by solid sheets of pleomorphic epithelioid tumour cells and brisk mitotic activity. The papillary growth architecture may be focally retained [17] but can be completely absent [18]. Some CPCs are predominantly composed of small primitive neuroectodermal cells with a high nuclear‐to‐cytoplasmic ratio, reminiscent of medulloblastomas or other embryonal tumours [19]. The diagnosis of CPC requires at least four of the following five criteria: brisk mitotic activity (> 5 mitoses per 10 high‐power fields; 0.23 mm^2^), marked nuclear pleomorphism, high cellularity, loss or blurring of the papillary growth pattern with solid growth and necrosis. When adjacent CNS tissue is present, diffuse brain invasion is common.

Clinically, CPC demonstrates an aggressive course. Metastatic disease is present at diagnosis in 21% of patients [16], and despite multimodal treatment strategies, including maximal surgical resection combined with chemotherapy and radiotherapy, the clinical behaviour of CPC is variable, with 5‐year OS rates between 56% and 64% [20, 21]. In addition, many long‐term survivors experience treatment‐related neurocognitive sequelae [22].

## 3 Challenges in the Grading of CPTs

The histological grading of CPTs can be subjective, particularly with regard to the more qualitative criteria such as cellular density and nuclear pleomorphism. In practice, grading is straightforward at the two ends of the spectrum: benign CPP with a low mitotic count and very low proliferative activity and overtly malignant CPC with exceedingly high mitotic activity and a predominantly solid growth pattern. The principal difficulty arises in the intermediate range, where a tumour with papillary architecture shows increased mitotic activity together with additional atypical features, rendering the distinction of aCPP from CPC challenging.

Two considerations help to mitigate this variability. First, grading should be anchored to the most reproducible and prognostically robust criterion, the mitotic count, which was the only atypical feature independently associated with recurrence on multivariate analysis [9]. In the same series, the 24 CPCs showed a mean mitotic activity of 13 mitoses per 10 high‐power fields, and only three had fewer than five mitoses per 10 high‐power fields [9]. Although an aCPP may occasionally reach a high mitotic count, it would be unusual for a CPC to fulfil the additional (and more subjective) features of malignancy while showing a low mitotic count, which helps to resolve many borderline cases. Second, molecular assessment can support the diagnosis, as CPCs almost invariably fall into the high‐risk methylation group (see next section); a tumour interpreted as CPC on histology but assigned to a low‐risk methylation group should therefore prompt reconsideration of the diagnosis. In difficult or borderline cases, review with experienced colleagues or a reference centre, complemented by ancillary molecular profiling, provides a robust basis for the final grade.

## 4 Molecular Pathology of CPTs

### 4.1 Genetic Alterations

Even though the majority of CPTs occur sporadically, CPCs are strongly associated with *Li*–*Fraumeni* syndrome (OMIM #151623), a classic cancer predisposition disorder caused by pathogenic germline variants in the *TP53* tumour suppressor gene [23]. In children presenting with CPC, the detection rate of pathogenic germline *TP53* variants is estimated to be 40%–50% [24, 25]. An exception is a region in southern Brazil, where (due to a founder effect) more than 60% of CPC patients harbour a specific *TP53* germline variant (NM_000546.6: c.1010G > A, p.Arg337His, ClinVar #12379) [26]. In addition to germline alterations, somatic *TP53* variants have been reported in 36%–60% of CPCs [25, 27, 28], whereas they occur only rarely (approximately 5%) in CPP [25]. Importantly, *TP53* mutation status also has prognostic relevance, as patients with *TP53*‐wild‐type CPC exhibit significantly longer progression‐free and overall survival than those with *TP53*‐mutant tumours [25, 29]. Therefore, *TP53* sequencing of tumour tissue is highly informative in the neuropathological investigation of CPC. Furthermore, given the high prevalence of pathogenic *TP53* germline variants in this tumour entity, genetic counselling and germline *TP53* sequencing are recommended in all CPC patients, irrespective of family history or somatic *TP53* status, to enable identification of Li–Fraumeni syndrome and to guide appropriate clinical surveillance [23].

Other rare constitutional disorders have been reported in association with CPTs. However, these associations appear considerably weaker than those observed for Li–Fraumeni syndrome. Aicardi syndrome (OMIM #304050), a presumed X‐linked neurodevelopmental disorder characterised by agenesis of the corpus callosum, chorioretinal lacunae and infantile spasms, has been linked to the development of CPPs, occasionally with multifocal presentation. CPP has also been described in patients with hypomelanosis of Ito, a neurocutaneous mosaic disorder (OMIM #300337) frequently associated with chromosomal abnormalities, including reported cases with a t(X;17) translocation [30, 31] and loss of heterozygosity on chromosome 10 [32]. In addition, rare cases of CPP have been reported in individuals with constitutional 9p duplication syndrome [33].

Whole‐exome and whole‐genome sequencing studies of CPTs have identified few recurrent single‐nucleotide variants beyond *TP53* [29, 34, 35, 36]. In CPPs of adult patients, *TERT* promoter hotspot mutations (C228T and C250T) occur in 25% and have been associated with shorter progression‐free survival [35]. In *TP53*‐wild‐type CPCs, additional somatic variants have been reported in genes such as *ERCC2*, *JAK2* and *EPHA7* [35, 36]; however, these alterations are non‐recurrent, and their role in CPC tumourigenesis remains unclear.

At the chromosomal level, aneuploidy is a characteristic feature of CPTs and can provide a useful diagnostic clue. Most CPTs harbour multiple chromosomal copy‐number alterations, resulting in markedly aneuploid genomes. However, methylation array–derived copy‐number profiles may misassign the diploid baseline in highly aneuploid tumours, leading to ploidy estimates that differ from sequencing‐based approaches [37]. Near‐diploid or flat copy‐number profiles are observed in fewer than 5% of cases and should prompt careful re‐evaluation of the diagnosis, particularly in the differential diagnosis with non‐neoplastic choroid plexus and in distinguishing CPC from atypical teratoid/rhabdoid tumour [27, 35]. Within this generally aneuploid genomic landscape, distinct patterns of chromosomal imbalance have been observed across CPT subtypes: cytogenetic and array‐based investigations demonstrated mainly hyperdiploid genomes in CPPs and aCPPs, whereas CPCs display either hypodiploid or hyperdiploid chromosome sets [27, 35, 38, 39, 40, 41]. Most hyperdiploid CPCs are characterised by acquired uniparental disomy of several chromosomes, with chromosome 17 (containing *TP53*) being most affected by this alteration [27]. Certain chromosomal alterations have also been associated with clinical outcome: gain of chromosome 9p and loss of chromosome 10q were associated with longer survival in patients with CPCs [40], whereas loss of chromosome 12q was linked to worse prognosis [42]. However, the biological significance of these chromosomal alterations remains poorly understood, and it is currently unclear whether they arise through stepwise genomic evolution or result from a single catastrophic chromosomal event during tumour development.

### 4.2 Epigenetic Characteristics

DNA methylation–based classification of CNS tumours has emerged as a highly robust tool to improve diagnostic accuracy and prognostication [43, 44]. In CPTs, methylation analysis identifies three principal methylation classes, and with the v12.8 release of the Heidelberg Brain Tumour Classifier, an additional fourth subclass has been incorporated, the biological and clinical significance of which remains to be fully characterised [44].

Table 1 summarises the different nomenclatures used for CPT methylation subgroups. Tumours assigned to the ‘Paediatric Subtype A’ cluster largely comprise CPPs and aCPPs occurring in young children, typically arising in supratentorial locations. In contrast, tumours of the ‘Adult Subtype A’ cluster mainly represent infratentorial CPPs and aCPPs of adult patients, and *TERT* promoter mutations are predominantly confined to this subgroup. CPCs are invariably classified as ‘Paediatric Subtype B’; however, this epigenetic subclass also contains a proportion of CPPs and aCPPs. Of note, aCPPs of the ‘Paediatric Subtype B’ subclass show a higher probability to recur as compared to aCPPs of the methylation subclasses ‘Paediatric Subtype A’ and ‘Adult Subtype A’ [41, 46]. Furthermore, the methylation subclass ‘Paediatric Subtype B’ appears to be epigenetically heterogeneous [47]. Consistent with this observation, the v12.8 release of the Heidelberg Brain Tumour Classifier identified a novel subgroup enriched for CPCs in adult patients, although the clinical relevance of this subgroup requires validation in larger clinicopathological cohorts [44].

**TABLE 1:** Nomenclature, histopathology, clinical and genetic features of epigenetic choroid plexus tumour subgroups.

Designation v12.8 CNS tumour classifier [44]	Paediatric Subtype A	Adult Subtype A	Paediatric Subtype B	Adult Subtype B
Designation in [41]	Cluster 1	Cluster 2	Cluster 3	
Histopathology [45]	CPP (59%) aCPP (41%)	CPP (79%) aCPP (21%)	CPP (10%) aCPP (17%) CPC (73%)	Unknown
Age group	Paediatric	Adult	Paediatric	Adult
Genetic findings [35]	*TERT/TP53* wild type	*TERTp* mut (33%)	*TP53* mut (42%)	Unknown
Tumour location	Supratentorial	Infratentorial	Supratentorial	Supratentorial

In general, CPP and aCPP belonging to the ‘Subtype A’ methylation classes are associated with a relatively favourable (‘low‐risk’) clinical course, whereas aCPPs assigned to ‘Subtype B’ show more aggressive biological behaviour. Importantly, mutational testing adds a layer of risk stratification, as *TERT* promoter mutations further stratify tumours within the ‘Adult Subtype A’ group, whereas *TP53* pathogenic variants identify a particularly high‐risk subset within the ‘Paediatric Subtype B’ class.

The three principal CPT methylation subgroups are reproducibly assigned across DKFZ classifier versions and alternative platforms [48, 49], supporting the technical robustness of subgroup assignment and the previously reported outcome correlations. However, further validation in independent, clinically homogeneous cohorts is warranted.

Taken together, histological grade remains the principal prognostic determinant in larger clinical cohorts [46]. Assignment of the molecular subclass ‘Paediatric Subtype B’ in a benign CPP is of unknown relevance. In contrast, aCPPs assigned to this methylation class carry an increased recurrence risk [41, 46] and may warrant closer follow‐up. This molecular finding does not alter the WHO grade but may be included as a comment in the neuropathology report.

## 5 Differential Diagnosis

**FIGURE 1:**
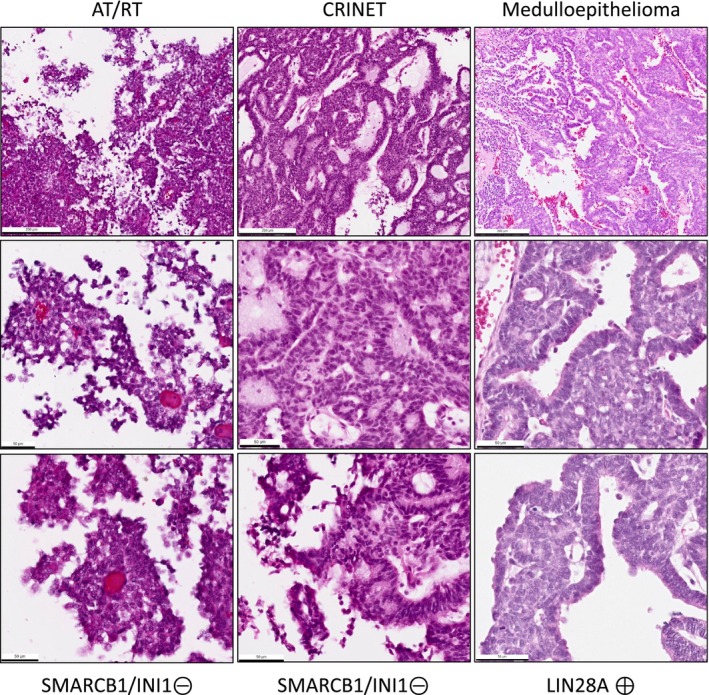
Histological differential diagnoses of choroid plexus tumours in infants. The principal differential diagnoses in this age group include atypical teratoid/rhabdoid tumour (AT/RT), cribriform neuroepithelial tumour (CRINET) and the medulloepithelioma variant of embryonal tumour with multilayered rosettes (ETMR). ⊕, positive; ⊝, negative/lost. All images are haematoxylin and eosin (H&E) stained sections. Corresponding whole slide images are available at https://omero‐imaging.uni‐muenster.de/webclient/img_detail/736925.

**FIGURE 2:**
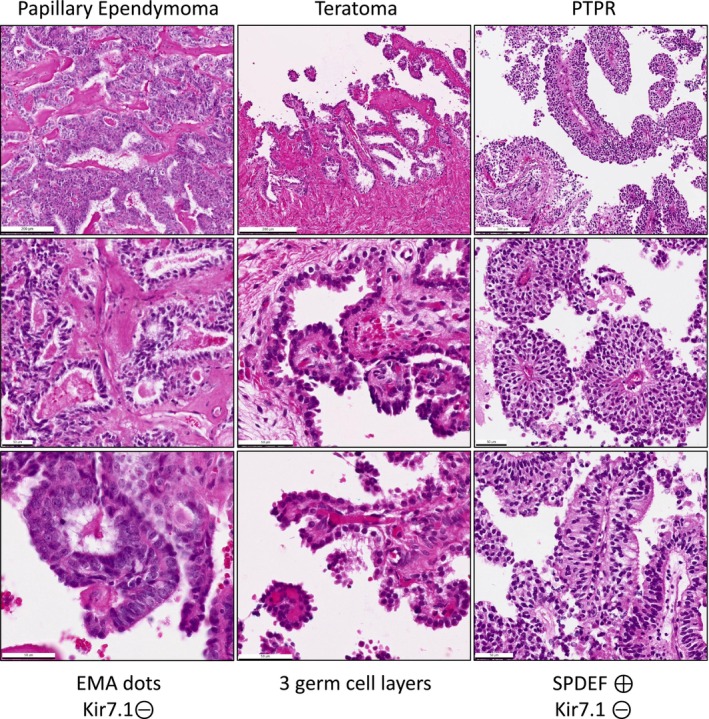
Histological differential diagnoses of choroid plexus tumours in children and adolescents. The principal differential diagnoses in this age group include papillary ependymoma, teratoma and papillary tumour of the pineal region (PTPR). ⊕, positive; ⊝, negative/lost. All images are haematoxylin and eosin (H&E) stained sections. Corresponding whole slide images are available at https://omero‐imaging.uni‐muenster.de/webclient/img_detail/736925.

**FIGURE 3:**
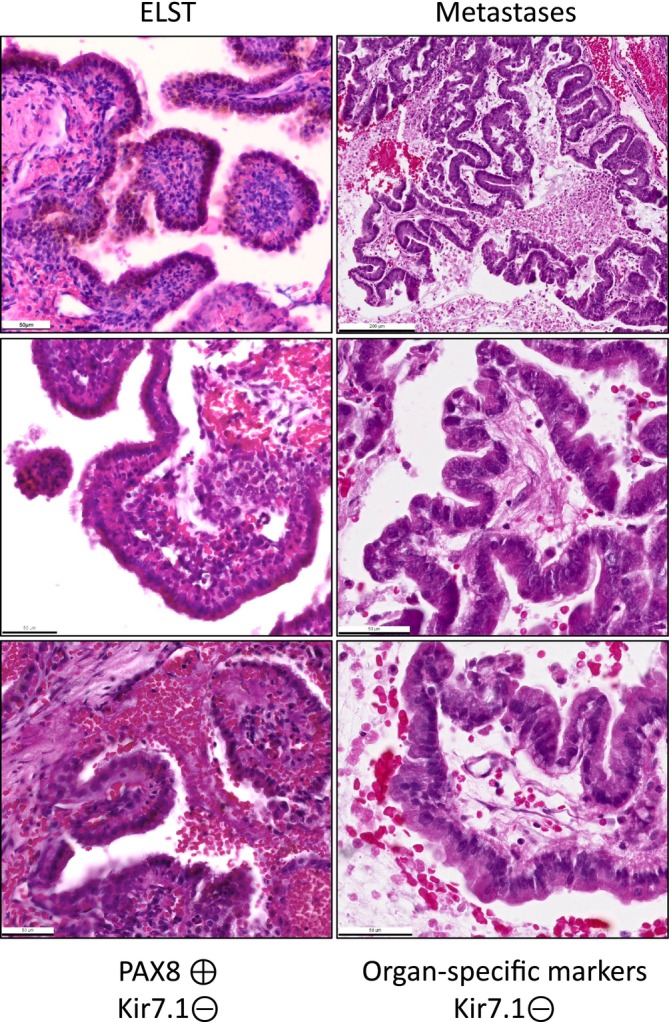
Histological differential diagnoses of choroid plexus tumours in adults. The key differential diagnoses in this age group are endolymphatic sac tumour (ELST) and intraventricular metastases. ⊕, positive; ⊝, negative/lost. All images are haematoxylin and eosin (H&E) stained sections. Corresponding whole slide images are available at https://omero‐imaging.uni‐muenster.de/webclient/img_detail/736925.

The differential diagnosis of CPTs includes a heterogeneous group of neoplasms that may share morphological features such as papillary architecture and intraventricular location. Because many of these entities show distinct age predilections, integration of the clinical context, particularly the patient's age, can substantially narrow the range of relevant diagnostic considerations. In the following sections, the most important differential diagnoses are discussed according to the age groups in which these tumours most commonly arise, focusing on lesions encountered in infants, children and adults (Figures 1, 2, 3).

### 5.1 Infants

#### 5.1.1 Atypical Teratoid/Rhabdoid Tumour (AT/RT)

AT/RT is the most critical differential diagnosis to consider when evaluating malignant CNS tumours with epithelial features in infants. AT/RT predominantly affects children younger than 3 years, with a peak incidence below 1 year of age [50], and carries a particularly poor prognosis; the 5‐year overall survival of approximately 35% [51] compares unfavourably with that of CPC. Histologically, AT/RT is characterised by variable morphology, encompassing not only rhabdoid cells with vesicular chromatin, prominent nucleoli and eosinophilic globular cytoplasmic inclusions but also poorly differentiated small round blue cells as well as areas of mesenchymal or epithelial differentiation [52] (Figure 1; whole slide image: https://omero‐imaging.uni‐muenster.de/webclient/img_detail/736925/). The immunohistochemical profiles of CPC and AT/RT may also overlap, with shared expression of vimentin, cytokeratins and GFAP [53]. An important anatomical clue is tumour location: Whereas CPC in children arises almost exclusively supratentorially, with the lateral ventricle representing the predominant site [8], AT/RT with epithelial differentiation shows no such predilection and frequently involves the posterior fossa [52]. Rarely, AT/RT may also present as an intraventricular mass [54]. The epithelial pattern, which is most prone to mimicking CPC, is strongly associated with the AT/RT‐TYR molecular subgroup (specificity 97%), which also shows the highest rates of cytokeratin positivity [52]. Adding further complexity, a subset of AT/RTs focally expresses Kir7.1 [53], a membranous potassium channel initially considered specific for choroid plexus epithelium [55], and some cases also co‐express transthyretin, both markers previously thought to support a CPC diagnosis [53]. The molecular hallmark of AT/RT is biallelic inactivation of *SMARCB1*, resulting in loss of nuclear SMARCB1/INI1 expression, which is absent in true CPC and represents the single most important ancillary diagnostic marker [56]. In addition, DNA methylation analysis reliably separates AT/RT from CPT, with *SMARCB1*‐deficient AT/RTs clustering in distinct methylation classes (AT/RT‐TYR, AT/RT‐SHH, AT/RT‐MYC) that do not overlap with CPT methylation subgroups [44].

#### 5.1.2 Cribriform Neuroepithelial Tumour (CRINET)

Cribriform neuroepithelial tumour (CRINET) is a rare intraventricular neoplasm first described in 2009 and characterised by a cribriform epithelial architecture and loss of SMARCB1/INI1 expression in the absence of rhabdoid cytomorphology typical of AT/RT [57] (Figure 1; whole slide image: https://omero‐imaging.uni‐muenster.de/webclient/img_detail/736928/). CRINETs arise in the vicinity of both supratentorial and infratentorial brain ventricles, predominantly in infants and young children [58]. Histologically, these tumours are composed of cribriform strands and ribbons of epithelial cells with small lumina, particularly evident in compact areas; rhabdoid tumour cells are absent. The columnar epithelial cells forming these cribriform structures may resemble the single‐layered cuboidal epithelium of the choroid plexus, and in combination with the frequently high proliferative activity (median Ki‐67 index approximately 30%), this appearance can lead to confusion with CPC [58]. Immunohistochemically, CRINET demonstrates loss of SMARCB1/INI1 expression and shows positivity for tyrosinase, a feature shared with the AT/RT‐TYR molecular subgroup [58]. In contrast to CPC, CRINET characteristically displays strong surface‐related EMA expression [57], which can provide an additional diagnostic clue alongside the loss of SMARCB1/INI1. At the molecular level, CRINETs harbour biallelic inactivation of *SMARCB1* and deletions of chromosome 22q. DNA methylation analysis places these tumours within the AT/RT‐TYR methylation class [58]. Despite these molecular similarities to AT/RT, CRINET exhibits a considerably more indolent clinical course. Most reported patients achieve long‐term survival following surgical resection, suggesting that CRINET may represent a biologically and clinically distinct entity within the spectrum of *SMARCB1*‐deficient tumours [58].

#### 5.1.3 Medulloepithelioma Variant of Embryonal Tumour With Multilayered Rosettes (ETMR)

Embryonal tumour with multilayered rosettes (ETMR) is a highly aggressive CNS neoplasm of early childhood, often characterised by amplification of the *C19MC* microRNA cluster on chromosome 19q13.42, predominantly affecting children under 4 years of age [59]. The medulloepithelioma variant of ETMR may arise within the ventricular system [60] and is particularly relevant in the differential diagnosis with CPC: These tumours are composed of primitive neuroepithelial cells arranged in tubular, papillary or trabecular structures resembling the embryonic neural tube (Figure 1; whole slide image: https://omero‐imaging.uni‐muenster.de/webclient/img_detail/736934/). These formations may superficially mimic the papillary architecture of CPC, particularly in small biopsy samples. However, the primitive cells typically display internal and external limiting membranes, reflecting true neuroepithelial differentiation, a feature not present in CPT. LIN28A immunoreactivity, a relatively sensitive and specific marker for ETMR, including the medulloepithelioma variant [59, 61], and expression of transthyretin and Kir7.1 in CPTs [55] can further support the differential diagnosis. Demonstration of C19MC amplification by FISH or next‐generation sequencing, which is absent in CPT, confirms the diagnosis of ETMR when present. However, a substantial proportion of ETMR, in particular *DICER1*‐mutant tumours, lack C19MC amplification. DNA methylation analysis, which assigns both C19MC‐altered and C19MC‐negative ETMR to a class entirely separate from CPT methylation subclasses, provides the most reliable distinction [44]. Given the markedly poorer prognosis of ETMR compared with CPC, accurate molecular classification is essential for appropriate treatment planning.

### 5.2 Children

#### 5.2.1 Papillary Ependymoma

Ependymal tumours are CNS neoplasms that originate from the wall of the ventricular system along the entire craniospinal axis. In the 2021 WHO classification of CNS tumours, papillary ependymoma is no longer recognised as a distinct subtype [1]. This change reflects the limited diagnostic specificity of papillary morphology. In a study of 19 tumours previously classified as papillary ependymoma, DNA methylation investigation confirmed an ependymoma diagnosis in only 63% of cases; one tumour was reclassified as CPPs and those with true papillary architecture predominantly mapped to the posterior fossa group B (PFB) methylation class [62]. Histologically, the key features distinguishing ependymoma from CPP are perivascular pseudorosettes (fibrillary cytoplasmic processes tapering towards vessel walls) and the absence of a continuous single‐layer columnar epithelium (Figure 2; whole slide image: https://omero‐imaging.uni‐muenster.de/webclient/img_detail/736940/). Immunohistochemistry can further aid the distinction. Ependymomas typically express GFAP and demonstrate a characteristic dot‐like epithelial membrane antigen (EMA) staining pattern corresponding to intracytoplasmic microlumina [63]. This dot‐like pattern contrasts with CPTs, which frequently show no EMA expression at all or at most weak and focal staining [64]. In morphologically ambiguous cases, DNA methylation assessment provides definitive classification and reliably separates ependymoma from CPT methylation classes [44, 62].

#### 5.2.2 Teratoma

Intracranial teratomas predominantly arise in the first two decades of life and show a predilection for the pineal and suprasellar regions; intraventricular locations are occasionally encountered [65]. Mature and immature teratomas enter the differential diagnosis of CPT when they contain papillary epithelial components derived from the ectodermal germ layer (Figure 2; whole slide image: https://omero‐imaging.uni‐muenster.de/webclient/img_detail/736946/). The defining feature of teratoma is the simultaneous presence of tissues from all three germ layers, that is, ectoderm, mesoderm and endoderm, within the same neoplasm, a finding that is by definition absent in CPT. Immature teratoma additionally contains primitive neuroepithelial elements resembling fetal tissue that may suggest a high‐grade primary neuroepithelial tumour. Rarely, tumours with predominantly primitive or mesenchymal components may show only limited areas of choroid plexus–like differentiation, which can complicate the distinction from immature teratoma or other embryonal neoplasms. An isolated case described as choroid plexus blastoma showed blastematous and mesenchymal components with only minor recognisable choroid plexus epithelium [66]. In that report, definitive classification was achieved by DNA methylation testing, which assigned the tumour to the ‘Paediatric Subgroup B’ methylation subgroup of CPT. In addition to intracranial teratomas representing differential diagnoses for CPTs, extracranial gonadal and extragonadal teratomas may rarely contain CPTs arising within the teratomatous tissue, mainly in cases with CPP and aCPP morphology [67, 68, 69].

Immunohistochemistry provides useful ancillary support in the differential diagnosis. Markers such as OCT4, alpha‐fetoprotein (AFP) and placental alkaline phosphatase (PLAP) may highlight specific germ cell tumour components. Elevated serum or cerebrospinal fluid levels of AFP or β‐hCG also support a diagnosis of germ cell tumour and are not seen in CPT. In morphologically challenging cases, DNA methylation investigation assigns CPTs and germ cell tumours to clearly distinct methylation classes and provides definitive classification [44].

#### 5.2.3 Papillary Tumour of the Pineal Region (PTPR)

Papillary tumour of the pineal region (PTPR) is a rare epithelial‐like neoplasm arising in the pineal region and characterised by a loose papillary architecture composed of fibrovascular cores lined by layers of large cuboidal to columnar tumour cells with clear cytoplasm and basally located nuclei [70] (Figure 2; whole slide image: https://omero‐imaging.uni‐muenster.de/webclient/img_detail/736943/). Immunohistochemically, PTPR typically shows strong expression of cytokeratins, S100 protein, neuron‐specific enolase and vimentin, whereas EMA and GFAP are usually absent or only weakly expressed [70]. Based on its anatomical location, immunophenotype and ultrastructural features, the tumour is thought to arise from specialised ependymal cells of the subcommissural organ [70, 71]. PTPR may closely resemble CPP histologically, making immunohistochemistry an important adjunct in the differential diagnosis. In contrast to CPTs, which frequently express Kir7.1 and stanniocalcin‐1, most PTPRs lack immunoreactivity for these markers [72]. In addition, SPDEF immunohistochemistry may assist in this distinction, as SPDEF shows strong expression in the majority of PTPRs but is only rarely expressed in CPTs [71]. Recent molecular studies have demonstrated that PTPR can be subdivided into at least two epigenetic subgroups (A and B) with distinct clinical features: Subgroup A occurs predominantly in adults, whereas subgroup B tends to occur in younger patients and shows an association with pathogenic *PTEN* germline variants [71, 73, 74].

### 5.3 Adults

#### 5.3.1 Endolymphatic Sac Tumours (ELSTs)

Endolymphatic sac tumours (ELSTs) are papillary, slow‐growing neoplasms of the inner ear arising from the epithelium of the endolymphatic sac in the posterior petrous temporal bone (Figure 3; whole slide image: https://omero‐imaging.uni‐muenster.de/webclient/img_detail/736931/). They typically present as cerebellopontine angle or posterior fossa masses in adults, with a median age at diagnosis of approximately 31 years [75, 76]. Twenty‐three percent of cases occur in association with *von Hippel–Lindau* (VHL) disease, in which bilateral ELSTs are virtually pathognomonic and tend to arise at a younger age than sporadic tumours [77]. In both sporadic and *VHL*‐associated cases, tumourigenesis is driven by biallelic inactivation of the VHL gene [78]. Because CPP arising in the cerebellopontine angle can show a closely similar papillary architecture, ELST represents an important differential diagnosis for adult CPP in this location [79, 80]. Immunohistochemically, ELST typically shows nuclear PDGFR‐β expression in both epithelial and stromal components, epithelial EpCAM and EMA positivity, strong membranous cytokeratin staining and consistent PAX8 expression; in contrast, CPP characteristically demonstrates strong epithelial S‐100 protein expression, cytoplasmic vimentin staining and transthyretin positivity, features not seen in ELST [80]. In addition, Kir7.1 and EAAT‐1 have been reported as highly specific markers for CPP, being expressed in most CPPs but absent in ELST [81]. Metastatic clear cell renal cell carcinoma represents another important differential diagnosis, particularly in patients with VHL syndrome. Although both entities may express PAX8 and CAIX, ELST typically lacks RCC marker and CD10 expression, which favours its distinction from metastatic renal cell carcinoma [82]. Identification of pathogenic *VHL* alterations can provide further diagnostic support, as CPP has not been associated with *VHL* disease [35, 80].

#### 5.3.2 PTPR

PTPR should also be considered in the differential diagnosis of papillary tumours in adults, particularly for lesions arising in the posterior third ventricular or pineal region. Although rare overall, PTPR occurs predominantly in adolescents and adults [74] and may histologically resemble CPP because of its papillary architecture and epithelial immunophenotype as mentioned above.

#### 5.3.3 Intraventricular Metastases

Intraventricular or choroid plexus metastases may closely mimic CPC in adults and can present a diagnostic challenge (Figure 3; whole slide image: https://omero‐imaging.uni‐muenster.de/webclient/img_detail/736937/), particularly when the clinical history of a primary malignancy is unknown. Although CPC predominantly arises in infants and young children, it may rarely occur in adults, in whom it most often involves the lateral ventricle but can also arise in the fourth ventricle or cerebellopontine angle [83]. Consequently, metastatic carcinoma must be systematically excluded before a diagnosis of CPC is rendered in adult patients [84, 85]. Intraventricular metastases are uncommon, accounting for less than 5% of all intracranial metastases, and most frequently occur in the lateral ventricles [86, 87]. Large stereotactic radiosurgery series have shown that the most common primary tumours giving rise to intraventricular metastases include renal cell carcinoma, lung carcinoma, melanoma and breast carcinoma, whereas thyroid carcinoma is only rarely encountered but represents a particularly important differential diagnosis because its papillary architecture may closely resemble CPTs [86, 87]. Histologically, metastatic adenocarcinomas involving the choroid plexus may show papillary or glandular architecture with marked mitotic activity and necrosis, features that can closely overlap with those of CPC [88, 89]. Immunohistochemistry therefore plays a central role in the differential diagnosis. Organ‐specific markers such as TTF‐1 and napsin A (lung adenocarcinoma), CDX2 (colon carcinoma), GATA3 and oestrogen receptor (breast carcinoma), or RCC, PAX8 and CD10 (renal cell carcinoma) are absent in CPTs. Conversely, markers characteristic of choroid plexus epithelium, including Kir7.1 and stanniocalcin‐1, are typically expressed in CPTs but not in metastatic carcinomas [55]. Although the epigenetic profiles of metastases (or their primary) tumours are not incorporated in the Heidelberg Brain Tumour Classifier [44], they can be analysed using the crossNN classifier, which incorporates methylation classes from the TCGA consortium [48]. Both classifiers include CPTs, allowing reliable distinction from metastatic lesions in diagnostically challenging cases.

## 6 Conclusion

In conclusion, the differential diagnosis of CPTs encompasses a diverse spectrum of neoplasms that varies considerably by patient age. In infants, the principal diagnostic challenge lies in the distinction of CPC from AT/RT, CRINET and the medulloepithelioma variant of ETMR—entities that share epithelial features, intraventricular location and partially overlapping immunophenotypes. Retained nuclear SMARCB1/INI1 expression reliably separates CPC from AT/RT and CRINET, whereas absence of LIN28A expression and lack of C19MC amplification argue against ETMR. In children and adolescents, papillary ependymoma, teratoma and PTPR represent the most relevant differential diagnoses. Ependymoma is distinguished by perivascular pseudorosettes and a characteristic dot‐like EMA staining pattern, teratoma by the presence of tissues from multiple germ layers and expression of germ cell markers and PTPR by the absence of Kir7.1 together with frequent SPDEF positivity. In adults, tumours of the endolymphatic sac and PTPR may mimic CPP, whereas intraventricular metastases with papillary morphology represent the most important differential diagnosis of CPC and must be systematically excluded using organ‐specific immunohistochemical markers. In the majority of cases, the diagnosis of a CPT can be established based on histomorphology and a targeted immunohistochemical panel alone. However, when conventional assessment remains inconclusive, DNA methylation analysis can provide useful additional information; sub‐threshold classifier scores or erroneous subgroup assignments remain a recognised limitation, though the presence of widespread copy‐number alterations derived from methylation arrays constitutes an independent argument in favour of a CPT diagnosis. Methylation‐based classification additionally yields valuable prognostic information, as distinct epigenetic subgroups have been shown to correlate with clinical outcome. Beyond tumour classification, *TERT* promoter mutation in adult CPP and aCPP is a marker for increased recurrence risk. In addition, *TP53* sequencing is particularly important in CPCs, as the high rate of pathogenic *TP53* germline variants in this tumour type requires identifying patients who may have *Li‐Fraumeni* syndrome and need genetic counselling and long‐term surveillance.

## Author Contributions

Christian Thomas and Martin Hasselblatt conceived the review. Christian Thomas performed the literature review and drafted the manuscript. Martin Hasselblatt supervised the work and critically revised the manuscript for important intellectual content.

## Funding

This work was supported by Deutsche Krebshilfe (70117416).

## Ethics Statement

The use of anonymized biopsy specimens for research, including scanned histological slides made publicly available as part of this review, was conducted in accordance with local regulations of the University Hospital Münster and approved by the Münster ethics committee under approval numbers 2007‐420‐f‐S and 2017‐707‐f‐S. All specimens were obtained in a diagnostic context and were anonymized prior to research use.

## Conflicts of Interest

The authors declare no conflicts of interest.

## Data Availability

Data sharing not applicable to this article as no datasets were generated or analysed during the current study.
